# Effect of Chiral Damping on the dynamics of chiral domain walls and skyrmions

**DOI:** 10.1038/s41467-022-28815-6

**Published:** 2022-03-07

**Authors:** C. K. Safeer, Mohamed-Ali Nsibi, Jayshankar Nath, Mihai Sebastian Gabor, Haozhe Yang, Isabelle Joumard, Stephane Auffret, Gilles Gaudin, Ioan-Mihai Miron

**Affiliations:** 1 Univ. Grenoble Alpes CNRS, CEA, Grenoble INP, SPINTEC, Grenoble, France; 2 CIC nanoGUNE BRTA, 20018 Donostia-San Sebastian, Basque Country Spain; 3 Department of Physics, Clarendon Laboratory, University of Oxford, Oxford, United Kingdom; 4 C4S, Physics and Chemistry Department, Technical University of Cluj-Napoca, Cluj-Napoca, Romania

**Keywords:** Spintronics, Electronic devices

## Abstract

Friction plays an essential role in most physical processes that we experience in our everyday life. Examples range from our ability to walk or swim, to setting boundaries of speed and fuel efficiency of moving vehicles. In magnetic systems, the displacement of chiral domain walls (DW) and skyrmions (SK) by Spin Orbit Torques (SOT), is also prone to friction. Chiral damping (α_c_), the dissipative counterpart of the Dzyaloshinskii Moriya Interaction (DMI), plays a central role in these dynamics. Despite experimental observation, and numerous theoretical studies confirming its existence, the influence of chiral damping on DW and SK dynamics has remained elusive due to the difficulty of discriminating from DMI. Here we unveil the effect that α_c_ has on the flow motion of DWs and SKs driven by current and magnetic field. We use a static in-plane field to lift the chiral degeneracy. As the in-plane field is increased, the chiral asymmetry changes sign. When considered separately, neither DMI nor α_c_ can explain the sign reversal of the asymmetry, which we prove to be the result of their competing effects. Finally, numerical modelling unveils the non-linear nature of chiral dissipation and its critical role for the stabilization of moving SKs.

## Abstract (web summary)

Chiral damping plays a critical role in the motion of skyrmions and domain walls, but it difficult to distinguish its influence from Dzyaloshinskii Moriya Interaction (DMI). Here, Safeer et al show that competition between chiral damping and the DMI result in a sign change in the chiral asymmetry.

## Introduction

**Fig. 1:**
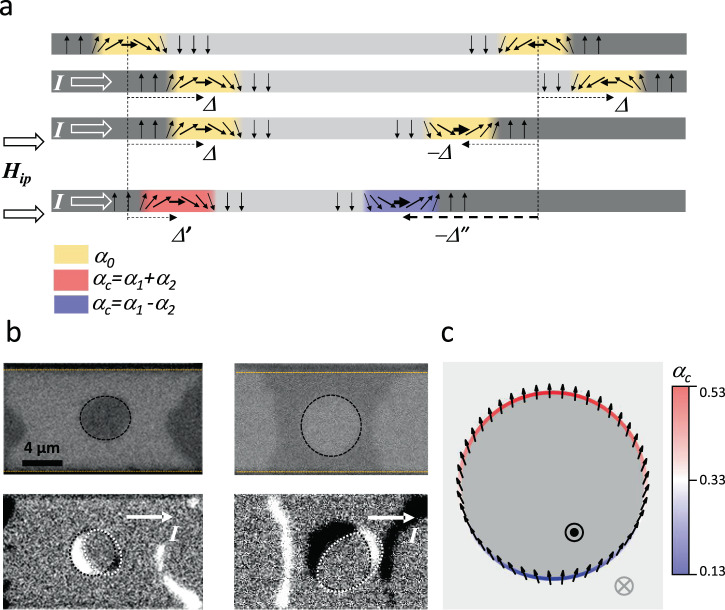
Effect of chiral damping on the DW and SK dynamics. **a**. Schematic representation of current-induced motion of chiral DWs by SOT. The DWs with the same chirality (set by DMI) are displaced by the same amount (Δ represented by the dotted arrows) and in the same direction by the current induced SOT. When an external in-plane field sets the magnetization of the two DWs in the same direction, it imposes opposite chiralities. In this case, the two DWs still move by the same amount, but in opposite directions (Δ and −Δ). In the presence of chiral damping, the two DWs with different chirality experience different damping (red and blue). This engenders different mobility, which causes the different magnitude of the opposite displacements (**Δ′** and **−Δ**ʺ). **b**. Kerr images of magnetic bubble motion driven by electric current pulses (5000 · 20 ns at 1·10^12^ A/cm^2^) in Pt/Co/Pt tri-layers. The two initial states correspond to bubbles magnetized either up (top-left) or down (top-right). The black dotted lines are guides for the eye indicating the initial bubble position. The orange dotted lines indicate the lateral edge of the magnetic pad. As seen in the differential Kerr images (below), the bubbles are displaced along the direction of the electric current (white arrow), but also undergo a chiral distortion (from circular to elliptical; the orientation of the ellipse depends on the chirality). The white dotted lines indicate the final state of the bubble. **c**. DW magnetization profile (black arrows) along the bubble perimeter under the effect of DMI and horizontally applied electric current (H_DMI_ = 200 mT; the damping-like and field-like components of SOT are H_FL_ = 33 mT and H_DL_= 66 mT). The dark/bright grey indicates the up/down magnetization. This image is to illustrate that as the chirality changes along the perimeter of the bubble, the value of the chiral damping (color scale) will also vary, leading to an angular-dependent DW mobility.

The observation of DMI^1,2^ in metallic multilayers has allowed significant advances in understanding the mechanism of the current induced DW motion^3–6^, as well as the development of the current induced SK dynamics^7–14^. These phenomena, potentially useful for applications are closely related. Their efficiency relies on two pillars: efficient SOT and strong DMI. In materials with broken inversion symmetry and with large spin-orbit interaction, the electric current produces a damping like torque (DL)-SOT^15,16^, and the DMI effective field, deriving from a chiral energy contribution, imposes a Néel DW structure^4^. In this configuration, the magnetization of the DWs is parallel to the current (Fig. 1a), which makes the SOT most efficient in producing DW displacements.

By analogy with DMI, which is a chirality-dependent energy, the chiral damping is a chirality-dependent dissipation. Several theoretical approaches have successfully predicted different possible origins, as well as diverse manifestations^17–22^. The main feature though, is that in practice, as the chirality is modified, the damping coefficient changes. A simple way to express this for the case of a DW in a perpendicularly magnetized material of interest here, is ${{{{{{\rm{\alpha }}}}}}}_{c}={{{{{{\rm{\alpha }}}}}}}_{1}+{{{{{{\rm{\alpha }}}}}}}_{2} ({\vec{{{{{{\rm{m}}}}}}}}_{{ip}}{{{{{\rm{\cdot }}}}}}\nabla {\vec{{{{{{\rm{m}}}}}}}}_{z})$. Here ${\vec{{{{{{\rm{m}}}}}}}}_{{ip}}{{{{{\rm{\cdot }}}}}}\nabla {\vec{{{{{{\rm{m}}}}}}}}_{z}$ is the projection of the in-plane DW magnetization (${\vec{{{{{{\rm{m}}}}}}}}_{{ip}}$) on the normal to the DW position ($\nabla {\vec{{{{{{\rm{m}}}}}}}}_{z}$) (Figs. 1a, c). Because this projection can be negative, α_1_ must be larger than α_2_ to ensure that the damping always remains positive.

Having the same symmetry and possibly driven by the same microscopic interactions, the effects of α_c_ cannot be easily separated from those of DMI. The ability to isolate them experimentally is crucial for the fundamental understanding of chiral phenomena in magnetism^23^, as well as from the purely pragmatic perspective of applications. Dissipative processes are fundamental to enable high performance, energy efficient, computing and storage devices based on magnetic SKs, and chiral DWs^24–26^.

In this work, we separate the respective contributions of DMI and α_c_ on the dynamics of DWs and skyrmionic bubbles and prove that the two phenomena compete (Fig. 1a). In particular, α_c_ modulates the DW and SK velocity and modifies the shape of the SK bubbles during their motion (Fig. 1b), affecting their dynamical stability^13,27^.

## Results and discussion

As a material platform for our study, we employ aPt_3 nm_/Co_0.6 nm_/Pt_1.5 nm_ tri-layer. The first reason is that in this material we have already observed evidence of chiral damping^17^. The second reason is that DMI is relatively weak, and allows controlling the chirality by moderate in-plane magnetic fields (H_ip_).

We study the chiral dynamics in the flow regime, by measuring the displacements of DWs and SKs induced by nanosecond current or out-of-plane field pulses (see methods) using wide-field Kerr microscopy. Our study covers all possible types of chiral dynamics: field-induced DW motion (FIDM), current-induced DW motion (CIDM), and current-induced SK motion (CISKM). These dynamics are very different: FIDM can be either turbulent or steady motion, the CIDM has a single highly non-linear regime, while the CISKM includes 2D effects, not present in the case of CIDM. The chiral effects are extracted by monitoring the asymmetric motion of DWs with opposite polarity (up/down and down/up). When applying a sufficiently strong H_ip_, as the magnetization of both DWs aligns with the field, their chirality becomes opposite (Fig. 1a). In this situation, the two DWs will have different energy (due to DMI) and different damping (due to α_c_). This reveals the competing effects of DMI and α_c_ on the DW motion asymmetry in all experiments.

Our analysis is supported by a numerical model, based on a *q-ϕ* approach, (see supplementary information S1). The physical parameters used in the simulations are either measured independently or obtained from fitting the experimental data. Because the experimental features outnumber the free variables in the model, the parameters are uniquely determined (Supplementary information S1). The simulations reproduce accurately the ensemble of our experimental results, by using the same set of values for all cases. Micromagnetic simulations based on MuMax^28^ (Supplementary information S10) are further used to assess the influence of α_c_ on the SK stability.

For the sake of clarity, in sections I, II, and III we present separately the three different experiments. In section IV, we present theoretical predictions of the model, using parameter values required for applications.

### I) Current induced DW motion

**Fig. 2:**
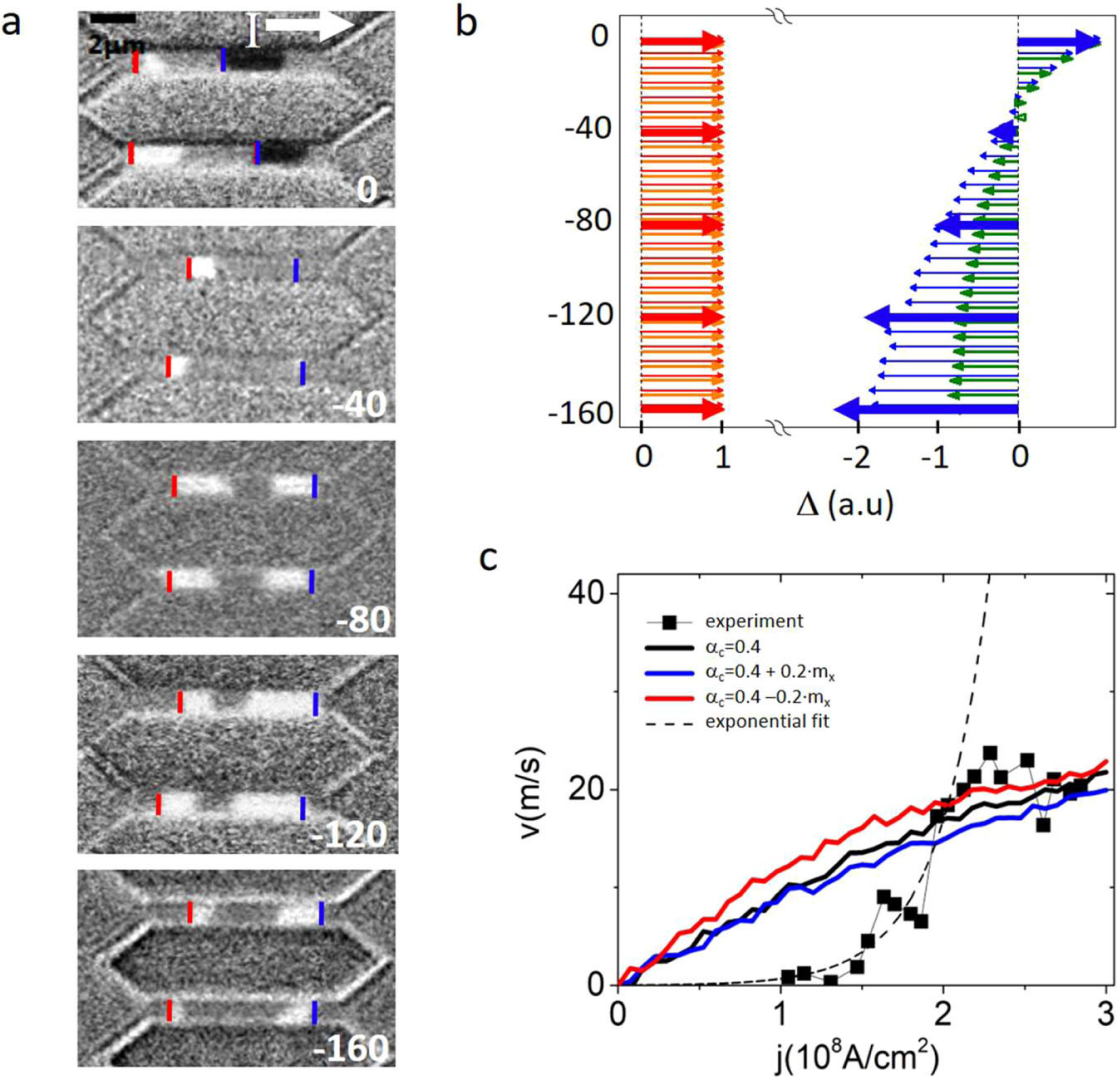
Current induced DW motion. **a**. Differential Kerr images showing CIDM for different values of H_ip_, shown in white (values in mT) on each image. Dark and white contrast indicates that up/down and down/up DWs are displaced in the same direction. Red and blue lines mark the starting positions for the DWs with opposite up/down polarities. The current density is fixed at 1·10^8^A/cm^2^. The pulse sequences used for each image are, from top to bottom: 10000 · 2 ns; 1000 · 1.5 ns; 100 · 1.5 ns; 200 · 1.2 ns; 300 · 1 ns **b**. Comparison of relative DW displacements extracted from the MOKE images (bold red and blue arrows) with numerical simulations. Since for every value of H_ip_ in the experiment we had to use a different pulse sequence, it is impossible to extract accurately the DW velocity. Therefore, to evidence chiral effects, we compare the respective displacement of the two DWs by normalizing to the value of the positive displacement (in red). Orange and green arrows correspond to the scenario of α_1_ = 0.4 and α_2_ = 0. Red and blue arrows correspond to α_1_ = 0.4 and α_2_ = 0.2. In the absence of chiral damping, the displacements at large H_ip_ become symmetric. The DWs move by the same amount in opposite directions. The reversal of the asymmetry observed experimentally, with the backward moving DW displacing a larger distance (blue arrows), is only reproduced in our model if we include the chiral damping. **c**. DW velocity as a function of current density (black symbols) for H_ip_ = 0. The numerical model reproduces well the data in the range of high current density, above 2·10^8^ A/cm^2^ and 20 m/s, within the boundaries of the flow regime. The dotted line is an exponential fit of the velocity in the intermediate range of current from 1·10^8^ A/cm^2^ to 2·10^8^ A/cm^2^. It is a guide for the eye to visualize the separation of the flow regime from the thermally activated DW motion.

The samples are patterned in the form of 10 µm long and 1 µm wide wires. The DWs are prepared using a perpendicular magnetic field. Under the effect of ns current pulses, the DWs shift in the direction of the current, indicating a left-handed DW chirality^4^. The DW velocity increases rapidly above 1·10^8^ A/cm^2^ (Fig. 2c). This rapid increase characteristic of the thermally activated DW motion stops above 2·10^8^A/cm^2^, signaling the limit of the flow regime. The saturation of the velocity observed above 2·10^8^A/cm^2^ is characteristic of the SOT-DMI mechanism in the flow regime, typically observed for materials with weak DMI^4^.

To evidence directly the chiral effects, we compare the displacements of up/down and down/up DWs as we apply H_ip_ (See Fig. S7 for the detailed Kerr images). The current density is fixed at 1· 10^8 ^A/cm^2^, sufficient to induce DW motion in the absence of H_ip_, but not to provoke nucleations at the largest H_ip_. Without H_ip_, both DWs move in the same direction, shifting the domain that they enclose (Fig. 2a) along the current flow. As H_ip_ is increased, the two DWs in the wire no longer move with the same velocity, leading to a contraction of the domain (Fig. 2a, b). This is because H_ip_ magnetizes them in the same direction. According to the DMI-SOT model it is expected that, as H_ip_ becomes much larger than H_DMI_, their motion should be symmetric^5,6^, without any net displacement of the central domain (Fig. 2b). Experimentally, the DWs indeed move symmetrically, but only at fields of approximately 80 mT. Above this value, the slow-moving DW (blue arrow) becomes faster (Fig. 2a, b), shifting the enclosed domain against the electric current. This is to say the unidirectional component of the motion has been reversed.

Though the DW motion at H_ip_ = 0 is hindered by pinning, the reversed unidirectional motion is observed at large velocity (>20 m/s) in the flow regime. Therefore, we can safely test the reversal of the unidirectional component of motion within our model. This result can only be reproduced by including both DMI and α_c_ (light blue and red arrows in Fig. 2b). In the absence of DMI there is no DW motion at H_ip_ = 0 (Fig. S5), and without α_c_, the DW motion at large H_ip_ is fully symmetric (orange and green arrows in Fig. 2b). The reason for this dependence is that at large H_ip_, the influence of H_DMI_ becomes negligible and the magnetization of the two DWs has the same orientation. Therefore, the effective field produced by SOT on the two DWs is identical. The reversal of the velocity asymmetry proves that DWs with opposite chirality have different damping. Thus, α_c_ is required to explain quantitatively and qualitatively the DW dynamics.

### II) Field-induced DW motion

**Fig. 3:**
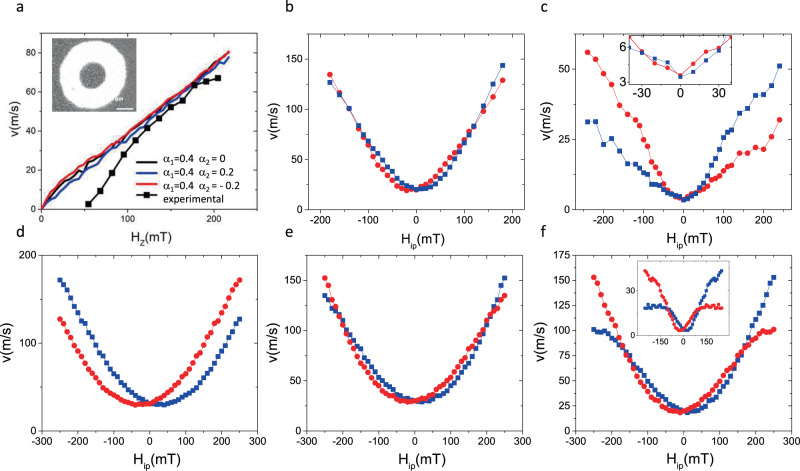
Field-induced DW motion. **a**. The experimentally measured DW velocity as a function of H_Z_ (black symbols). The lines are the results of the model with DMI only (black) or including α_c_ (blue and red). The model reproduces accurately the velocity data in the flow regime (the region with constant DW mobility). The inset shows a typical differential Kerr image. The bright ring represents the DW displacement produced by the field pulse. **b**. Measured DW velocity vs. H_ip_ (red for down/up DW, blue for up/down DW, see Fig. S8 for detailed Kerr images) at constant H_Z_ = −82 mT and **c**. H_Z_ = −55 mT. The inset is a zoom of the low field region where the crossing of two curves occurs. **d**, **e**, **f**, Calculated DW velocity with (d) H_Z_ = −82 mT and α_c_ = 0.4; (**e**) H_Z_ = −82 mT and α_c_ = 0.4 + 0.2·m_x_. (**f**) H_Z_ = −55 mT and inset H_Z_ = −10 mT.

We start by measuring the DW velocity vs. the out-of-plane magnetic field (H_Z_) without applying H_ip_ (Fig. 3a). We prepare bubble domains in an un-patterned film by applying a small H_Z_ field. To reach the flow DW motion and avoid the influence of pinning, we apply strong (up to −217 mT) but short (40 ns) H_Z_ pulses. The DW velocity (Fig. 3a) increases linearly from 40 m/s to 70 m/s when H_Z_ is larger than −110 mT, confirming the observation of the flow regime. For weaker H_Z_ values, the velocity drops fast, indicating a significant DW pinning below 40 m/s. In agreement with the low DW mobility, the numerical fitting of this curve reveals that the motion of the DW is turbulent. This is consistent with the low Walker breakdown field characteristic for materials with weak DMI^4^.

We then measure the asymmetry induced by H_ip_^17,29–31^ on the velocity of up/down and down/up DWs (See Fig. S8 for the detailed Kerr images). The effective in-plane field will be H_ip_ + H_DMI_ for down/up DWs, and H_ip_-H_DMI_ for up/down DWs. Therefore, the presence of DMI is recognized by a lateral shift of the velocity vs H_ip_ curves. To a first approximation, H_DMI_ can be extracted directly from the magnitude of the lateral shift^30,31^. The measured DW velocity (Fig. 3b) exhibits an almost parabolic dependence on H_ip_. The small lateral shifting of the curves indicates a small H_DMI_. Both the sign and magnitude of H_DMI_ are consistent with the CIDM experiment presented above. However, at large H_ip_ (>150 mT) there is a deviation from this behavior: as the velocity curves for the up/down (blue) and down/up (red) DWs cross, the DW motion asymmetry reverses. The fact that the asymmetry reversal occurs at large velocity (>100 m/s), where the DW motion is in the flow regime, allows us to apply our model to reproduce this experimental feature.

The simulations show that at low H_ip_, where DW motion is turbulent, because of the periodic changes of the chirality, α_c_ has little effect on the DW velocity. As H_ip_ exceeds a critical value required to stabilize the DW magnetization the DW periodic transformations cease. In this second regime of steady motion, the effect of chiral damping on the asymmetry is stronger than the opposing effect of DMI, leading to the change in sign. Indeed, if α_1_ = 0.4 and α_1_ = 0, in the steady regime the curves approach asymptotically but the crossing never occurs.

A simple way to verify this scenario experimentally is to repeat the measurements at weaker H_z_. In this case, a smaller H_ip_ should be sufficient to restore the steady DW motion and achieve the asymmetry reversal. Indeed, the velocity curves measured at H_Z_ = −55 mT exhibit a reversed asymmetry over an extended interval (Fig. 3c), starting at H_ip_ = 40 mT, while the positive asymmetry region is nearly suppressed. Once again, the model reproduces this tendency (Fig. 3f). Because the DW motion at H_Z_ = −55 mT starts to be affected by pinning, which is not included in the model, a better match between model and experiment is obtained using H_Z_ = −10 mT in the calculation (inset of Fig. 3f). This confirms that the reversal of the velocity asymmetry is caused by α_c_ and occurs as the DW motion becomes steady at large H_ip_.

### III) Current induced bubble deformation

**Fig. 4:**
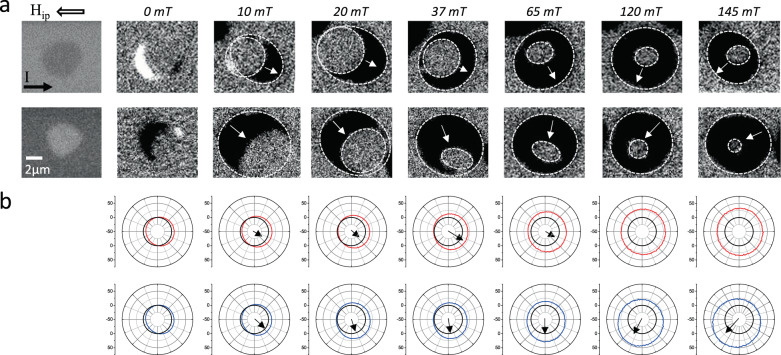
Chiral distortion of skyrmionic bubbles. **a**. Kerr images of the bubble displacement under the effect of current pulses (20 ns at 1·10^8^A/cm^2^) and H_ip_. On the first row we show images of a bubble magnetized up, on the second row, a bubble magnetized down. The first column shows the direct image of the initial bubble prepared by applying H_Z_. The rest of the columns show differential images of displacements induced by the current for different values of H_ip_ (from left to right: 0, 10, 20, 37, 65, 120, 145 mT). White dotted lines indicate the contours of the initial and final bubble. The white arrows point towards the direction of bubble displacement. The number of pulses is different for each image (top: 5000; 30,000; 10,000; 2000; 1500; 360; 120; bottom: 20,000; 50,000; 2500; 5000; 400; 100; 100;). **b**. Polar plot of computed DW velocity as a function of the angle between the DW orientation and electric current, corresponding to the same current density and H_ip_ values as the experimental data. In the top row, we show in red results from a scenario with DMI but without α_c_. The bottom row shows in blue results including both α_c_ and DMI. From the velocity dependence, we extract the direction of bubble displacement indicated by the black arrows. They are in agreement with the white arrows on the MOKE images that indicate the experimental bubble displacement.

Our next step is to evidence the effect of chiral damping on the current induced motion of the SK bubbles. For these experiments, the layers are patterned into 10 µm wide wires, large enough to contain the displacements of a single bubble (Fig. 4a). The bubbles can undergo plastic deformations, as they are not stabilized by the dipolar energy or DMI, but by local pinning. In this sense, they differ from stable SKs, but they have an important advantage: the shape resulting from the deformation is stable over time. Therefore, it is possible to measure the current-induced distortion of the bubbles. Such effects cannot be evidenced in stable SKs, which would return to their equilibrium position after the current is removed.

The experiments involving SKs also exhibit the features that we have observed in the CIDM experiments present above: *i)* bubbles are initially shifted in the direction of the current; *ii)* as H_ip_ increases, the bubble growth (shrinking) becomes predominant over the shifting; *iii)* at the largest H_ip_ (>80 mT) the shift is reversed, and the bubbles displace in the opposite direction.

The 2D geometry employed here allows to evidence two additional features. First, at zero H_ip_ the motion is asymmetric with respect to the angle between the current and the DW, leading to a distortion of the initially circular bubble towards an elliptical shape. Second, when H_ip_ is applied, the bubble not only shifts parallel to the current, it also shifts in the perpendicular direction.

These distorted bubble displacements stem from the specific angular dependence of the DW velocity with respect to the electric current^32–34^. To verify whether these observations are consistent with our understanding of the FIDM and CIDM, we use the 1D model to calculate the velocity as a function of this angle. The shifting of the bubbles (white arrows in Fig. 4a) is consistent with the asymmetry of the DW velocity predicted by the model (black arrows in Fig. 4b), only if α_c_ is included. This shows that α_c_ is fully responsible for the bubble distortion observed at large H_ip_.

### IV) Model predictions

**Fig. 5:**
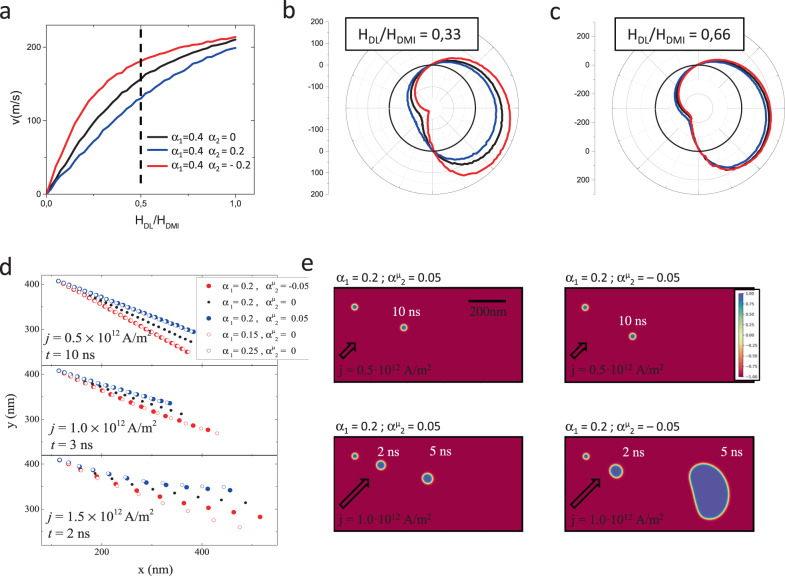
Modelling of the SK distortion in presence of the chiral damping. **a** CIDM plotted as a function of the ratio of H_DL_/H_DMI_. The dotted line approximately separates the linear variation regime from the plateau. Here, H_DMI_ = 0.2 T and H_DL_ = 2·H_FL_. The values chosen for these simulations are close to the optimal values and very importantly, they are realistic (typical for Pt/Co/AlO_x_ systems). **b** angular dependence of the velocity in the linear regime **c**. angular dependence of the velocity in the saturation regime. (black: α_1_ = 0.4; blue: α_1_ = 0.4, α_2_  = 0.2; red: α_1_ = 0.4, α_2_ = −0.2) **d**. Skyrmion position as a function of time for different current and damping values. **e** Superposed images of the out-of-plane component of the micromagnetic structure at different moments of time during the simulation. On each image, the SK on the left corresponds to the initial state. At low current density (top row) the SK structure remains intact during the displacement. Chiral damping only influences the trajectory. At larger currents, at first, the SK undergoes a slight distortion, which in the case of α_1_ = 0.2 and α^µ^_2_ = −0.05, leads to its instability and the SK explodes. Here the electric current is applied at 45° with respect to the stripe major axis.

Up to now, we have shown that all our experiments can be understood by including simultaneously the effects of both α_c_ and DMI. Moreover, the successful fitting of the entire dataset has allowed to establish numerical values for the different parameters (see Methods). However, one should be cautious when further using these values. They are not real values, but rather “effective values” which are model dependent in the sense that they incorporate all the approximations made in the model. For example, we neglect the second-order uniaxial anisotropy, the even components of chiral damping^21^ as well as the corrections to the gyromagnetic ratio^19,22^, we use an effective DW width; the temperature variation of the parameters with Joule heating is not considered (saturation magnetization, uniaxial anisotropy, DMI, SOT etc.). Therefore, these values should not be used to assess the strength of the microscopic interactions causing α_c_ and DMI. Nevertheless, since the model is explaining well all our experiments, we can use it to distinguish the roles played in the dynamics by each individual parameter (Fig. S5). Most importantly, we can also predict how these dynamics will evolve as these values change. In particular, we can focus on the range of values required for practical applications. From this perspective, there are two main requirements:

i. In order to stabilize the rigid Néel DW structure or small-size SKs, the DMI has to be much stronger than in the case of our experiments. The ceiling value for H_DMI_ is imposed by the spontaneous formation of helical phases, which obstructs the stabilization of single DWs or SKs^4^.
ii. Efficient current-induced motion requires larger DL-SOT compared to the Pt/Co/Pt layers used here, but not too large compared to H_DMI_. In this sense, Fig. 5a shows a calculation of DW velocity as a function of current density expressed as H_DL_/H_DMI_. The dependence can be approximately divided into two parts: in the low current regime, DW velocity increases with current, while in the second part, it reaches a plateau. The best efficiency of the CIDM is achieved at intermediate current (H_DL_/H_DMI_ < 0.5) where the velocity is large, but not saturated.

The numerical calculations indicate that the influence of the chiral damping will become stronger, as the DL-SOT and DMI approach the optimal values for applications (Fig. 5b, c). The angular dependence of the DW velocity (black curve in Fig. 5b, c), will tend to distort the SK circular structure affecting its dynamical stability. The chiral damping will reduce the distortion and improve the stability (blue curve in Fig. 5b, c). On the contrary, if α_2_ changes sign, it will accentuate the SK deformation (red curve in Fig. 5b, c). To understand the effect of the deformation on the SK stability, we perform micromagnetic simulations (S10), including chiral damping. We observe (Fig. 5e) that as long as the effect of current is insufficient to destabilize the SK structure, α_c_ modifies the SK velocity, but its influence on the anisotropic velocity is concealed by the rigidity of the magnetic structure. However, at larger driving force, close to the SK instability, the anisotropic velocity becomes relevant: α_c_ can either limit or enhance the SK distortion, influencing its stability. NB. The values of the damping coefficient used in the simulations are intentionally smaller than those of the 1D model. This is imposed by our simplified numerical integration of the chiral damping in the MuMax, which becomes approximate for larger damping. Moreover, the definition of the micromagnetic chiral component of the damping (α^µ^_c_) differs by a factor of 2 from its definition in the *q-ϕ* model (see Supplementary information S10).

Furthermore, the simulations of the SK trajectories (Fig. 5d), allow to evidence the effects of α_c_ by order of importance:

1^st^ order: The effects of damping can be observed in the linear response regime, where the driving force does not modify the chirality of the magnetic texture significantly. In this case, α_c_, can be approximated by a constant value that depends on the chirality (Fig. 5d). Note that it is very different compared to the Gilbert damping, which does not depend on the chirality. In a given material, the Gilbert damping coefficient is the same, regardless of the type of dynamics (uniform mode precession, SK motion etc…), while in presence of α_c_ the effective damping depends on the chirality of the magnetic texture involved in the dynamics.

2^nd^ order: As the driving force increases, the chiral texture distorts, and α_c_ creates *non-linear damping* (Fig. 5e). The SK trajectories deviate significantly from those obtained using a constant value. In this case, the damping varies in time and space (along the SK perimeter), and the evolution of the micromagnetic structure cannot be reproduced using a constant damping.

In conclusion, by performing a full set of experiments of DW and SK dynamics in Pt/Co/Pt, we were able to observe how chiral dissipation influences the DW motion in the flow regime, and also to understand how it affects the distortion of skyrmionic bubbles. In all experiments, we observe a reversal of the chiral asymmetry when an in-plane field is applied. The numerical model reproduces this behavior only if both α_c_ and DMI are included. In this case, all experimental features are comprehensively reproduced using a unique set of physical parameters. By extrapolating our numerical model to the range of large SOT and strong DMI, which is more suitable for applications, we evidence a strong influence of α_c_ on the SK dynamics and stability. Understanding and controlling α_c_ is thus essential for engineering devices employing DWs or SKs.

## Methods

### FIDM

We prepare DW bubbles in the unpatterned film by applying a small perpendicular field (5 mT for 1 s). Then we apply nanosecond field pulses (up to 217 mT for 40 ns) and extract the velocity from the DW displacements. Because of its small size and of the short pulses, the magnetic field of the micro-coil cannot be measured directly. It is calibrated by comparing low field DW velocity produced by the micro-coil to low field DW velocity produced by a macroscopic, calibrated coil.

### CIDM

In order to achieve large current densities, we pattern the samples into micrometer-sized wires. The DWs are prepared using a perpendicular magnetic field. Nanosecond current pulses with current densities ranging from 0.1·10^8 ^A/cm^2^ to 2·10^8 ^A/cm^2^ are used to propagate the DWs in the micro-wires (Fig. 2a). The velocity is determined at every current density from the linear dependence of the DW displacement vs pulse length.

When measuring the CIDM in presence of H_ip_, we cannot use the same procedure as described above. In these experiments, the Joule heating together with H_ip_ cause unwanted nucleations limiting the range of pulse amplitude and duration that we can use. Since the range of current pulses is reduced, we cannot quantify the DW velocity by the linear regression of the displacement vs. pulse length. Therefore, we have evidenced the chiral effects using the following procedure:

1. We set the current density to a value strong enough to produced DW motion for H_ip_ = 0, but not so strong to produce nucleations for the largest H_ip_ (145 mT).
2. The exact pulse width and pulse sequence (number and frequency) are optimized for every H_ip_ value in order to maximize the displacements, while avoiding nucleations.
3. As the current density remains constant, when H_ip_ is decreased, the number and frequency of pulses have to be increased in order to maintain the size of the measured displacements. This is a consequence of the weaker driving force provoking more frequent DW pinning to defects.

Using this procedure, we cannot quantify the velocity value as precisely as we did for the CIDM without H_ip_. For this reason, we compare the relative variation of the up/down and down/up DW displacements under identical conditions as we vary the chirality by H_ip_.

### Numerical model

The best agreement between the ensemble of experiments and the *q-ϕ* model was obtained with the following set of parameters: *DW width, Δ = 6* *nm; H*_*DMI*_* = 30 mT; DW dipolar field H*_*Dip*_* = 30 mT; α*_*1*_* = 0.4; α*_*2*_* = 0.2*.

## Supplementary information

supplementary information

Peer Review File

## Data Availability

The authors declare that all the data used to reach the conclusions, and necessary to reproduce the results, is presented in the manuscript and the supplementary information.

## Code availability

The code used to model the results is available from the corresponding author upon reasonable request.
