# Supplementary material for: Effect of Chiral Damping on the dynamics of chiral domain walls and skyrmions
Source: Nat Commun. 2022 Mar 7;13:1192. doi: 10.1038/s41467-022-28815-6 (PMC8901652; doi:10.1038/s41467-022-28815-6)
Supplement: Supplementary file 1 — supplementary information [file 41467_2022_28815_MOESM1_ESM.docx]

**Supplementary information**

**Effect of Chiral Damping on the dynamics of chiral domain walls and skyrmions**

C.K. Safeer^1,2,3^, Mohamed-Ali Nsibi^1^, Jayshankar Nath^1^, Mihai Sebastian Gabor^4^, Haozhe Yang^1^, Isabelle Joumard^1^, Stephane Auffret^1^, Gilles Gaudin^1^, & Ioan-Mihai Miron^1^*

*^1^Univ. Grenoble Alpes CNRS, CEA, Grenoble INP, SPINTEC, Grenoble, France*

*^2^CIC nanoGUNE BRTA, 20018 Donostia-San Sebastian, Basque Country, Spain.*

*^3^Department of Physics, Clarendon Laboratory, University of Oxford, Oxford, United Kingdom*

*^4^C4S, Physics and Chemistry Department, Technical University of Cluj-Napoca, Cluj-Napoca, Romania*

* To whom correspondence should be addressed.

E-mail: [mihai.miron@cea.fr](mailto:mihai.miron@cea.fr)

**S1. Numerical model**

**S2. Influence of H_DMI_**

**S3. Influence of α_c_**

**S4. Dipolar field of the DW**

**S5. Temperature**

**S6. Effect of H_ip_ on the DW motion asymmetry**

**S7. Limit of the 1D model for 2D dynamics: the local approximation**

**S8. MOKE images of CIDM in nanowires in presence of H_ip_**

**S9. MOKE images of FIDM of a bubble in presence of H_ip_**

**S10. Micromagnetic simulations**

**S1. Numerical model**

The DW motion at large driving forces is independent of material imperfections. For this reason, since the complexity of dealing with pinning and de-pinning effects is removed, this motion regime can be modelled more easily. Analytical models have been used successfully for materials with in-plane and out-of-plane anisotropy. Two motion regimes have been identified: steady DW motion, occurring at relatively low DW velocity, and turbulent motion at larger fields. In the steady regime, the DW structure is increasingly distorted as the velocity increases. Beyond a critical velocity, the DW internal structure is no longer stationary and transforms periodically.

In materials with perpendicular anisotropy, such as the ones used in the present study, the DW dynamics can be modeled by a few equations^1–3^.

The field driven DW velocity in the steady regime:

$v_{\mathrm{steady}} = \frac{\gamma\Delta}{\alpha}H_{z}$ (1)

in the turbulent regime:

$v_{turbulent} = \frac{\alpha\gamma\Delta}{1+\alpha^{2}}H_{z}$ (2)

The Walker critical field separating the two regimes:

$H_{W} \approx$ $\alpha\left( H_{DMI}+1/2 H_{Dip} \right)$ (3)

The current driven DW velocity in the SOT-DMI model^4^:

$v_{j} =\frac{\gamma\Delta H_{DMI}}{\sqrt{1+\left( C_{o}\cdot\frac{\alpha}{\Delta} \frac{H_{DMI}}{{j\cdot\xi}_{DL-SOT}} \right)^{2}}}$ (4)

With:

$v_{turbulent} = \frac{\alpha\gamma\Delta}{1+\alpha^{2}}H_{z}$ (5)

Here, γ is the gyromagnetic ratio; α is the damping; Δ is the DW width ; H_DMI_ is the DMI field; H_Dip_ is the dipolar field associated to the Néel DW structure; C_0_ is a constant depending on physical constants and sample parameters; *j* is the current density; ξ_DL-SOT_ is the efficiency of the damping like component of the SOT; H_ip_ is the external in-plane field, H_K_ is the anisotropy field of the sample. We note that the dynamics are determined by a relatively small number of parameters.

For the modelling of the DW motion, due to the complexity of our experiments, which include both magnetic fields and electric current, applied simultaneously at different angles, the numerical modelling is more convenient than analytical calculations. For this, we have chosen to model the DW motion using a collective coordinates *q-φ* approach^7^. This has the advantage of providing accurate results for cases where analytical solutions may be too complex. To ensure the validity of our numerical model, we have tested it for the simpler cases with well-known analytical results described by the above equations.

Such numerical models have been widely used to study the DW dynamics. Their advantage over micromagnetic simulations is that the computation time is much faster. At the same time, they yield the same physical results in the vast majority of situations^7^. The reason for this is that the approximation that they rely on, that of a rigid DW as a solitonic quasiparticle, is sufficient in most instances.

Moreover, since they rely on effective parameters, the results can be interpreted easily, and the influence of each physical parameter can be identified directly.

Our numerical approach includes both the chiral energy and chiral damping.

Chiral energy appears as an effective magnetic field acting on the core magnetization of the DW.

It has been shown theoretically that the chiral damping can take different forms: it can have a high-order dependence^8^ on *m_x_* and it can even affect the gyromagnetic ratio^5^. Our model only uses the simplest form (α_c_=α_1_+α_2_·m_x_), which is sufficient to account for all the chiral features seen in our experiments. We note that while higher order contributions may exist and can affect the dynamics, disentangling the second order effects, or the different forms of chiral damping is beyond the purpose of our present work.

Non-chiral parameters such as the current induced torques, the DW dipolar field, domain wall width are included. The effect of disorder is included in the form of temperature fluctuations.

The current induced torques as well as the anisotropy field were determined experimentally using independent (second harmonic) measurements. ξ_DL-SOT_ = 0.017 (T/10^12^ A/m^2^), ξ_FL-SOT_ = 0.004 (T/10^12^ A/m^2^).

The values that fit best our results (Δ = 6 nm; H_DMI_ = 30 mT; H_Dip_ = 30 mT; α_c_ = 0.4+0.2·m_x_) are uniquely determined and allow to model simultaneously the entire range of different experiments. The exact values of the non-chiral parameters affect little the general shape of the curves and have a weak influence on the emergence of chiral features (S6, S7).

**The fitting procedure**

The model is based on 5 independent fitting parameters, 2 fixed measured parameters:

Independent: α_1_, α_2_, H_DMI_, Δ, H_Dip_.

Measured: ξ_DL-SOT_, ξ_FL-SOT_.

Equations (1) – (5) show that these are all the parameters that can influence the DW motion.

We fit the following experimental features:

- The value of the velocity saturation of the CIDM in the flow regime (Figure 2c)
- The value of the DW mobility in the flow regime of FIDM (Figure 3a)
- Reversal of the unidirectional component of the CIDM (Figure 2a, b)
- Reversal of the chiral asymmetry of the FIDM (Figure 3b, c, d, e, f)
- Asymmetry of the current-induced 2D bubble distortion produced by H_ip_ (Figure 4)

From previous measurements in the creep regime we can apply more constraints on the chiral damping and DMI. From the magnitude and the saturation field of the anti-symmetric component of the DW motion in the creep regime^6^, we had previously determined that

α_2_/α_1_ ≈ 0.5 and

H_DMI_ + H_Dip_ ≈ 50 mT.

When including these additional constraints, the total number of independent experimental features (7) exceeds the number of free parameters (5). This means that the values that we extract for these parameters are uniquely determined.

To determine their numerical values, we varied the values manually to improve the fit. After a few iterations, we converged toward a set of values that reproduce reasonably well all the different experiments simultaneously.

In order to illustrate the influence of each parameter on the dynamics, in the following we will show how the independent variations of each parameter affect the results.

**S2. Influence of H_DMI_**

H_DMI_ field models the effect of the DMI interaction on the DW magnetization. In a *q-φ* model, this field either points in the direction of DW motion, or is opposed to it, thereby favoring the Néel type DW configuration. It has a drastic effect on current induced DW motion, as it tends to align the magnetization with the electric current, thus making the DL-SOT more efficient in producing DW displacements (Figure S1a). Moreover, by stabilizing the DW structure, it also increases the H_W_ value (Figure S1b) for the field driven DW motion.


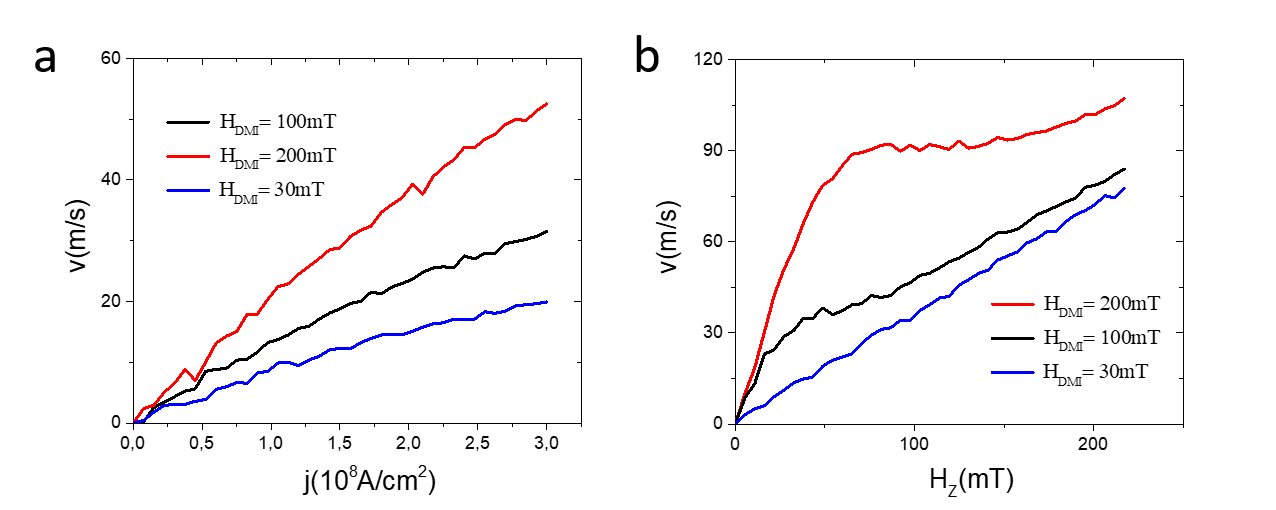


***Figure S1.*** ***a.*** *Current induced DW velocity,* ***b.*** *field induced DW velocity, calculated for three different H_DMI_ values.*

**S3. Influence of α_c_**

For the field induced DW motion in the turbulent regime, there is a periodic change of the chirality, thus canceling the effects of the chiral dependent damping.

In the case of current induced motion, the insensitivity to damping is due to the fact that the ratio H_DL-SOT_/H_DMI_ >1. This is seen directly in equation (4): when the H_DL-SOT_ is significantly larger than H_DMI_, as it is in Pt/Co/Pt tri-layers, the velocity becomes less sensitive to the damping value^4^.


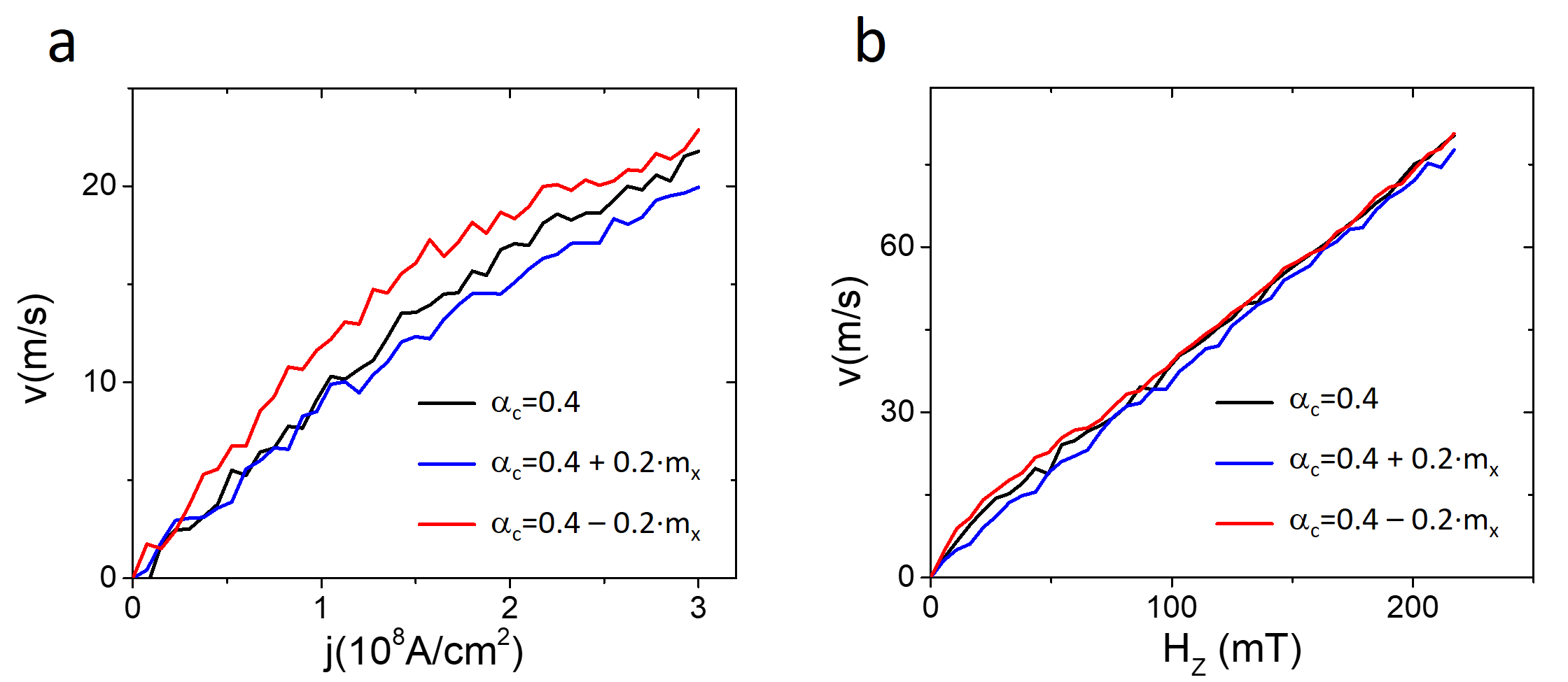


***Figure S2.*** ***a.*** *Current induced DW velocity,* ***b.*** *Field induced DW velocity, for different α_c_ values*

**S4. Dipolar field of the DW**

This effective field models the dipolar energy difference between the Bloch and Néel configurations. It will increase the stability of the Bloch DW structure and enhance the DW stability. Consequently, the increase of H_Dip_ will tend to lower the efficiency of the CIDM and to increase the Walker breakdown field.


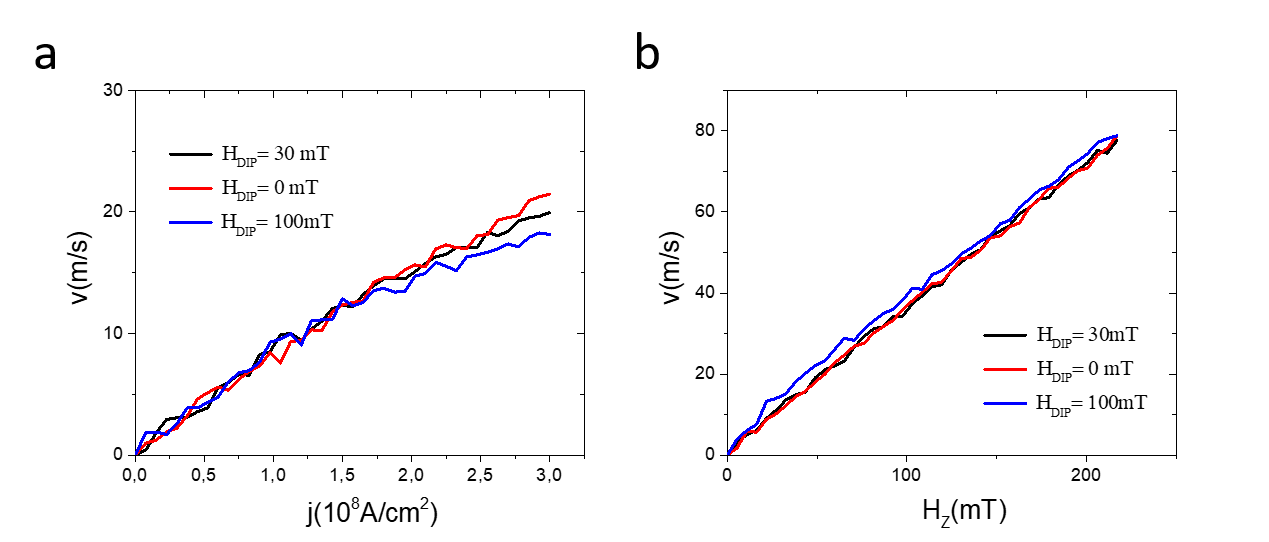


***Figure S3.*** ***a.*** *Current induced DW velocity,* ***b.*** *Field induced DW motion, for different H_Dip_*

**S5. Temperature**

By increasing the disorder, the temperature lowers the m_x_ component of the magnetization, thus reducing the efficiency of the CIDM. For the FIDM, the main effect of temperature is to smoothen the sharp features of the curves, such as Walker breakdown. Outside the range where the sharp features occur, the temperature value has little influence. The temperature is fixed at 300 K, while the “macrospin” volume subjected to temperature fluctuations is set to 0.5×6×10 nm for all the calculations. These dimensions correspond to the 0.5 nm = layer thickness, 6 nm= the DW width, and 10 nm = 2.5×*l_ex_* (the exchange length) an estimate of the length over which the thermal fluctuations do not disrupt significantly the parallel orientations of the spins^9^.


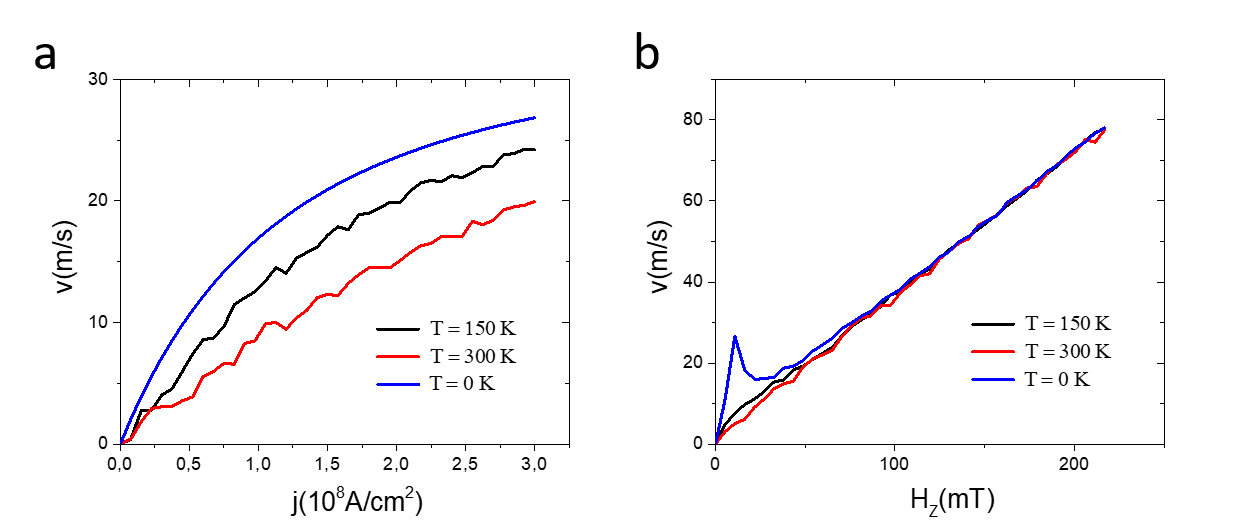


***Figure S4.*** ***a.*** *Current induced DW velocity,* ***b.*** *Field induced DW velocity for different temperatures*

**S6. Effect of H_ip_ on the DW motion asymmetry**

*a) FIDM*

First we analyse the field induced DW motion in absence of any chiral effects (Figure S6). H_ip_ increases the DW velocity as the motion passes from turbulent to steady. The velociy has an even dependence on H_ip_; the effects for the two DW polarities are identical. Moreover, H_ip_ has no effect on the steady motion regime.

The main effect of DMI is to compete with H_ip_, shifting the curves for the two polarities in opposite directions. It is only when we include the chiral damping that the velocity asymmetry changes sign in the steady regime. However, α_c_ alone can not produce a significant asymmetry in the turbulent regime. In order to reproduce the reversal of the asymmetry observed experimentally, it is required to include both α_c_ and DMI.

*b) CIDM*

In the absence of chiral effects, the velocity curves for the two DWs reflect the symmetry of the SOT: the DWs do not move at H_ip_ = 0; when H_ip_ ≠ 0 the two DWs move in opposite directions with the same velocity. The main effect of DMI is to shift the curves in opposite directions. Consequently, the DWs move even without H_ip_. However, when H_ip_ becomes sufficiently strong, the DWs still end up moving with the same velocity in opposite directions. In order to obtain different DW velocities at large H_ip_, we need to introduce α_c_. On the other hand, α_c_ alone cannot be responsible for the DW motion at zero field. Both α_c_ and DMI are required to reproduce the experimental observations.


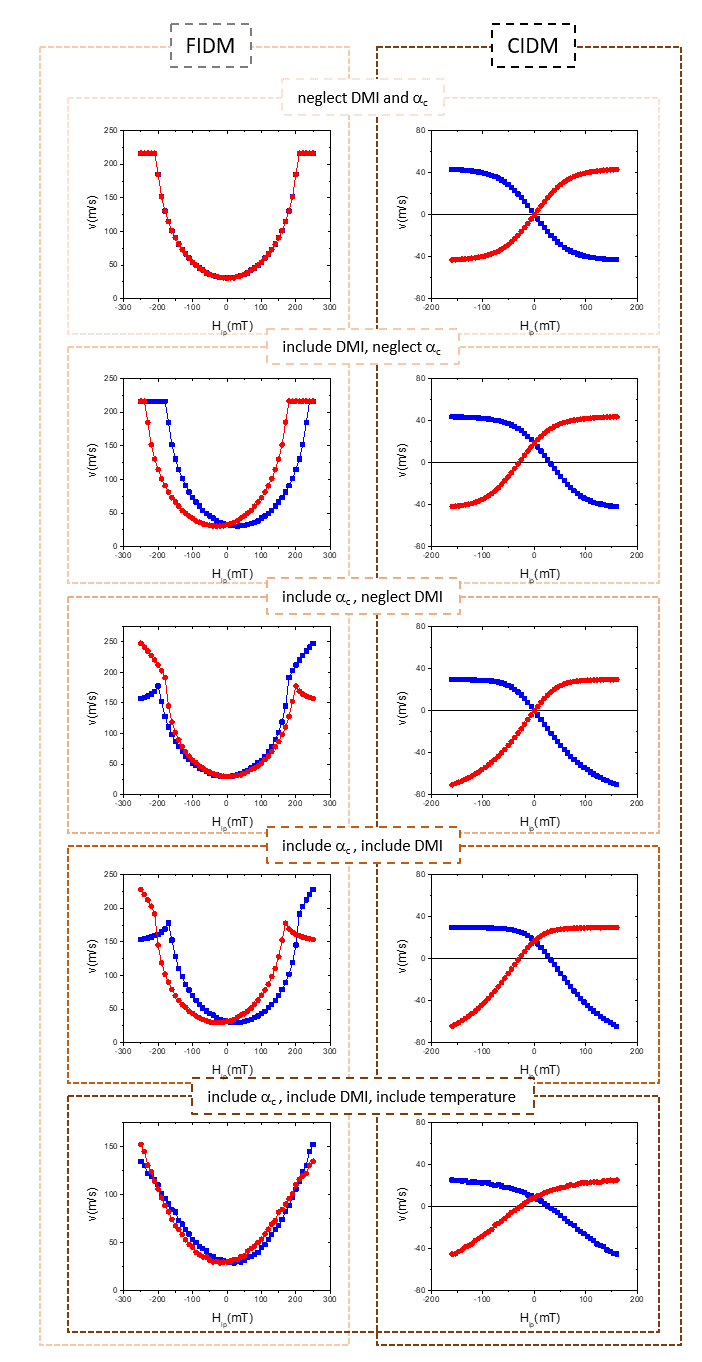


***Figure S5.*** *(left column) Effect of H_ip_ on field driven motion (H_Z_= 82 mT). We consider all the possible scenarios for the presence of chiral effects: no chiral damping and no DMI; only DMI; only chiral damping; both chiral damping and DMI. Temperature is also included. (right column) Effect of H_ip_ on current driven motion (j=10^8^A/cm^2^). We consider the same scenarios as for the field driven motion.*

**S7. Limit of the 1D model for 2D dynamics: the local approximation**

We have extended the use of the 1D model to study the motion and distortion of the 2D bubbles under magnetic field and current. For this we have performed 1D calculations for all the angles of the DW canting with respect to the current and the applied field, and from here we have reconstructed the angular dependence of the velocity vs. DW angle. This approach is not exact, because the DW behaves similarly to an elastic membrane so that the behavior at a given angle is influenced by the DW behavior at adjacent positions.

For this reason, the measured data must be considered with care when comparing with models.

Nevertheless, experimentally we observe that the bubbles can have complex shapes (Figure S6); the shape depends on the magnetic history and is weakly influenced by the DW elastic energy. This can be understood when considering that the bubbles are relatively large and the pinning relatively strong. When the pinning energy overcomes the elasticity, the bubbles can undergo plastic deformation, and the different sections of the bubble evolve almost independently of the rest. In this case, the 1D approximation is sufficient to describe their deformation.


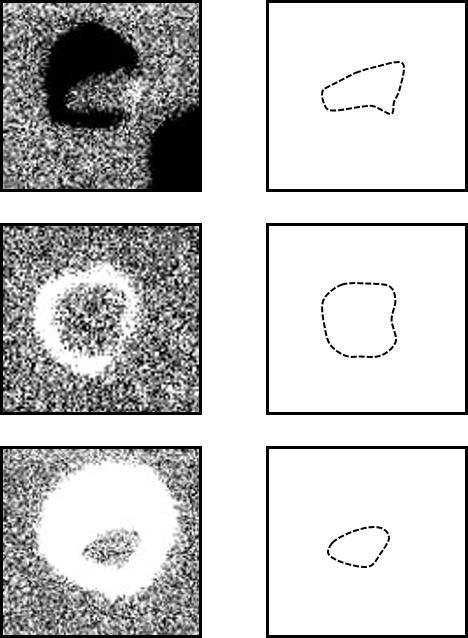


***Figure S6.*** *Differential Kerr images of bubble growth. The shape of the initial bubble is extracted from each image and depicted on the right column. The bubble can have vastly different shapes, depending on the precise sequence of magnetic field and current that was used to create it. This indicates an important plasticity of the bubble perimeter.*

**S8. MOKE images of CIDM in nanowires in presence of H_ip_**

**
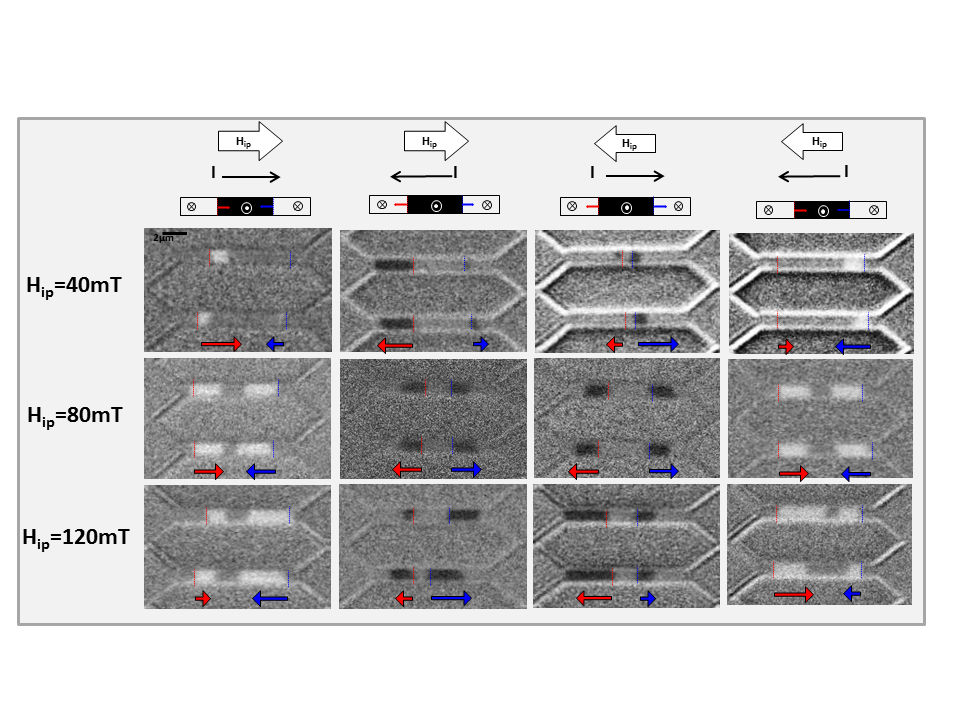
**

***Figure S7.*** *Differential Kerr images showing CIDM in the presence of H_ip_= 40 mT, 80 mT, and 120 mT for 4 different combinations of current and H_ip_ directions, depicted above the images. The motion of down/up and up/down DWs are schematically shown with red and blue arrows respectively. For each case, we clearly observe that the asymmetric DW motion at H_ip_= 40 mT along one direction becomes almost symmetric at H_ip_= 80 mT. At H_ip_= 120 mT, the DW motion becomes asymmetric again, but the asymmetry direction is opposite to that observed at H_ip_= 40 mT.*

**S9. MOKE images of FIDM of a bubble in presence of H_ip_**

**
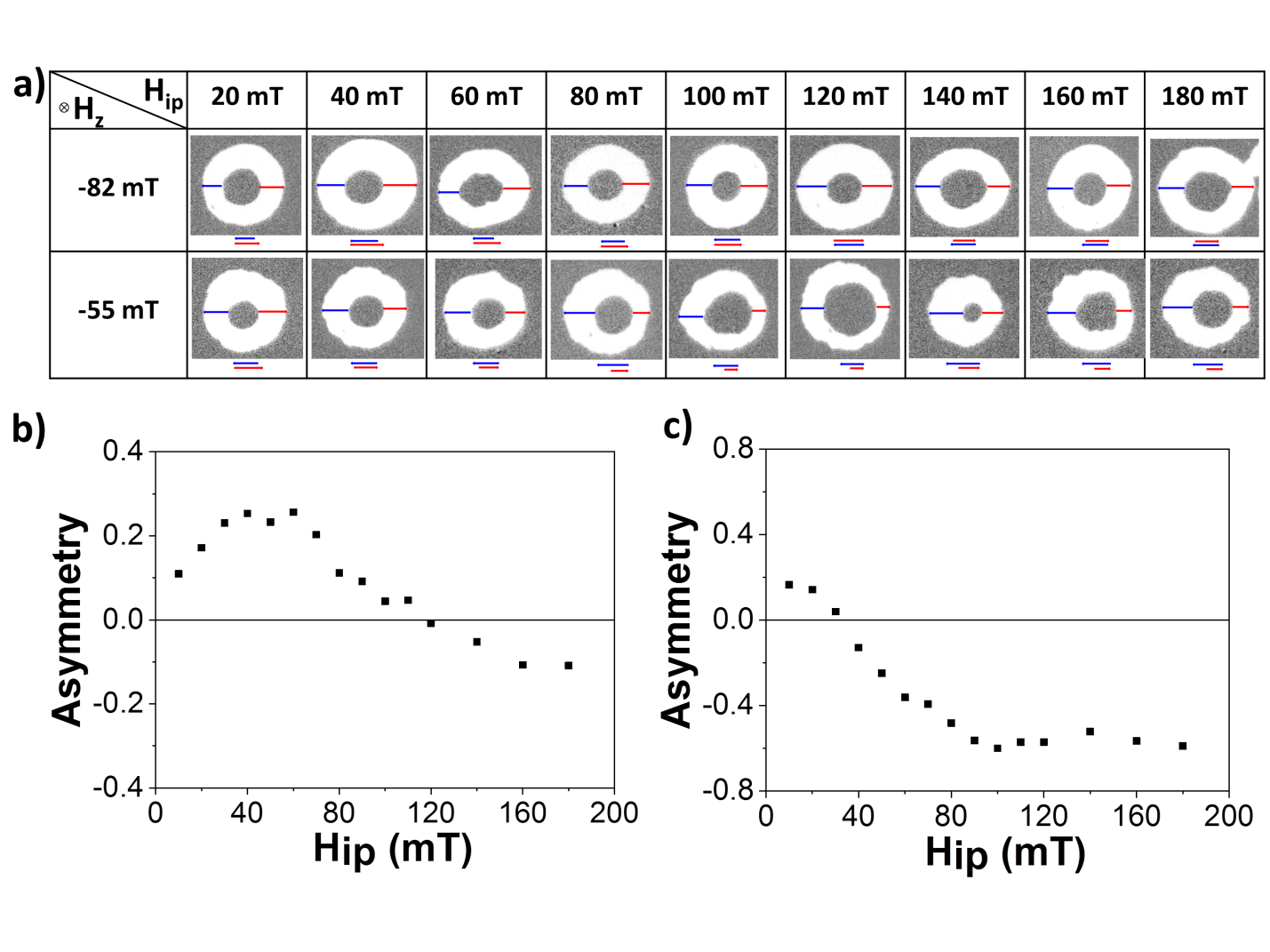
**

***Figure S8.*** ***a)*** *Differential Kerr images showing bubble expansion for H_z_= -82 mT and -55 mT for different values of H_ip_. The red and blue arrows schematically represent down/up and up/down DW displacements. The velocities extracted from these images were used in the plots shown in Figure 3 of the main text. Asymmetry=* $\frac{v_{down/up}-v_{up/down}}{{(v}_{down/up}+v_{up/down})/2}$*, calculated from the images* ***b)*** *at Hz= -82 mT* ***c)*** *at H_z_= -55 mT. For both cases, the asymmetry direction reverses as H_ip_ increases as explained in Figure 3 of the main text.*

**S10. Micromagnetic simulations**

The micromagnetic simulations were performed using the MuMax^3^ micromagnetic simulation software^10,11^. The chiral damping was implemented using the custom fields feature of MuMax^3^. As such, it is not included directly in the dissipative term, but rather as an effective field. The Landau–Lifshitz–Gilbert (LLG) governing the magnetization dynamics:

$$\frac{d\boldsymbol{m}}{dt}=-\gamma\boldsymbol{m}\times\boldsymbol{H}_{eff}+\alpha\boldsymbol{m}\times\frac{d\boldsymbol{m}}{dt}$$

can be transformed to:

$$\frac{d\boldsymbol{m}}{dt}=-\frac{\gamma}{1+\alpha^{2}}\boldsymbol{m}\times\left[ \boldsymbol{H}_{eff}+\alpha\left( \boldsymbol{m}\times\boldsymbol{H}_{eff} \right) \right]$$

where γ is the gyromagnetic ratio and $\boldsymbol{H}_{eff}=-\frac{1}{\mu_{0}M_{s}}\frac{\delta E}{\delta\boldsymbol{m}}$ is the effective field. Here, the energy includes the exchange, dipolar, anisotropy, DMI and SOT terms. Adding the chiral damping as an effective field is correct up to the renormalization of the gyromagnetic factor (1+α^2^)^-1^. Using this approximate implementation, we cannot model exactly the experimental results in our samples, because the experimental damping is too large for this approximation to be applicable. Nevertheless, this correction becomes negligible when the damping is significantly smaller than one (so α^2^ becomes negligible relative to unity). For this reason, in the micromagnetic simulations we use small values of damping. The comparison to experiments is thus not quantitative, but qualitatively correct. Including the chiral damping the LLG reads:

$$\frac{d\boldsymbol{m}}{dt}=-\frac{\gamma}{1+\alpha_{1}^{2}}\boldsymbol{m}\times\left[ \boldsymbol{H}_{eff}+\alpha_{1}\left( \boldsymbol{m}\times\boldsymbol{H}_{eff} \right)+\phi_{C}\left( \boldsymbol{m}\times\boldsymbol{H}_{eff} \right) \right]$$

where the last term in the brackets represents the *chiral damping effective field* and $\phi_{C}$ is given by^4^:

$$\phi_{C}={\alpha^{\mu}}_{2}\Delta\left[ m_{x}\frac{\partial m_{z}}{\partial x}-m_{z}\frac{\partial m_{x}}{\partial x}+m_{y}\frac{\partial m_{z}}{\partial y}-m_{z}\frac{\partial m_{y}}{\partial y} \right]$$

where we introduce a characteristic exchange length $\Delta=\sqrt{\frac{A}{K_{eff}}}$ with $A$ the exchange constant and $K_{eff}$ the effective anisotropy, and ${\alpha^{\mu}}_{2}$ the chiral component of the damping coefficient. Note that the micromagnetic definition of chiral damping is different from the *q - φ* model definition. In the *q - φ* model the chirality is expressed only with regard to the in-plane magnetization, while in the micromagnetic simulations the chirality is expressed both using the in-plane as well as the out-of-plane components. For this reason, equivalent results are obtained when the numerical value of chiral damping in the *q - φ* model is approximately a factor of 2 larger than the value used in the micromagnetic simulations.

For the simulations we used cells with a size of 1 nm. We chose realistic material parameters that were shown to stabilize skyrmions at room temperature in Pt/CoFeB/MgO layers^12^. Explicitly, the saturation magnetization $M_{s}=1.12\times{10}^{6} Am^{-1}$, perpendicular uniaxial anisotropy $K_{u1}={10}^{6} Jm^{-3}$, exchange constant $A={10\times10}^{-12} Jm^{-1}$, the DMI constant $D=1.5 mJm^{-2}$, the spin Hall angle $\theta_{SH}=0.07$ and the ratio between the field-like and the damping like SOTs $\xi=0.5$. During the SK simulations, an out-of-plane field of -10.4mT was applied.

Before studying the effect of chiral damping on the SK motion and their stability, we used the simulations to reproduce the main features of our experimental results. The goal is to validate the micromagnetic model that we will use further for studying the effect of chiral damping on the SK dynamics. It is to be noted that in this case we have used a lower DMI energy equivalent to a DMI field of $H_{DMI}=10.4 mT$. The simulations reproduce well all the characteristic features observed experimentally and exhibit a very good qualitative agreement with our *q-φ* model (Figures S9 and S10 compared to Figure S5). This is an important point because the micromagnetic simulations require fewer assumptions compared to the *q-φ* model. For example, they automatically account for the changes of the DW structure (such as the variation of the DW width) during the application of the electric current and the magnetic fields. Furthermore, to test the robustness of our conclusions, we also used different values of DMI and chiral damping (Figure S9, Figure S10). They confirm that the competition between DMI and chiral damping asymmetries is a general effect, occurring throughout the entire parameter space; the asymmetry reversal only occurs when the chiral damping is sufficiently strong to overcome the asymmetry produced by DMI.


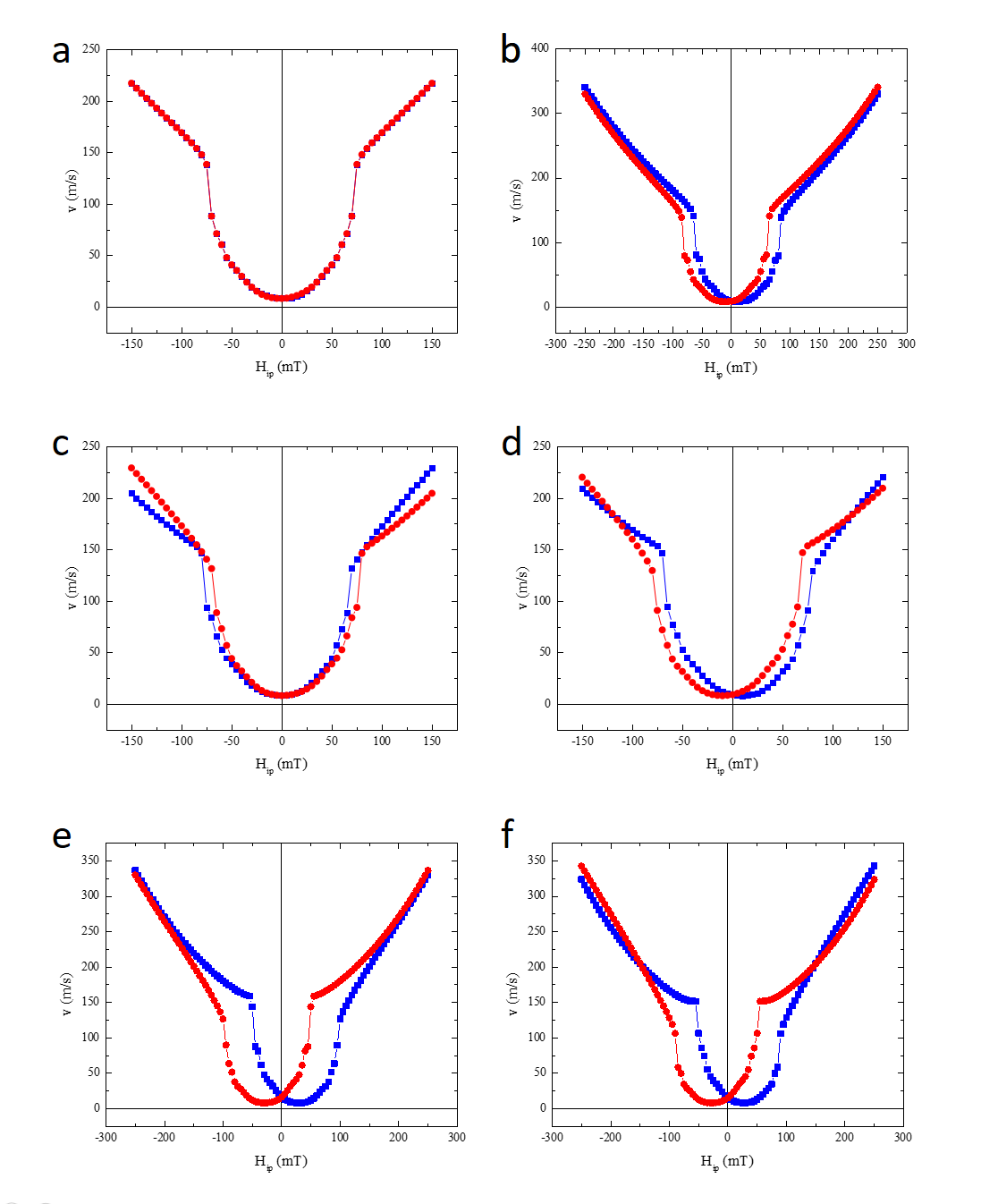


*Figure S9. Reversal of the asymmetry of the Field Induced DW motion (H_Z_ = 30 mT).* ***a****. H_DMI_ = 0, α_1_ = 0.2, α ^µ^_2_ = 0.* ***b****. H_DMI_= 10.4 mT, α_1_ = 0.2, α ^µ^_2_ = 0* ***c****. H_DMI_ = 0, α_1_ = 0.2, α ^µ^_2_ = 0.05.* ***d****. H_DMI_ = 10.4 mT, α_1_ = 0.2, α ^µ^_2_ = 0.05,* ***e****. H_DMI_ = 30 mT, α_1_ = 0.2, α ^µ^_2_ = 0.05,* ***f****. H_DMI_ = 30 mT, α_1_ = 0.2, α ^µ^_2_ = 0.1*


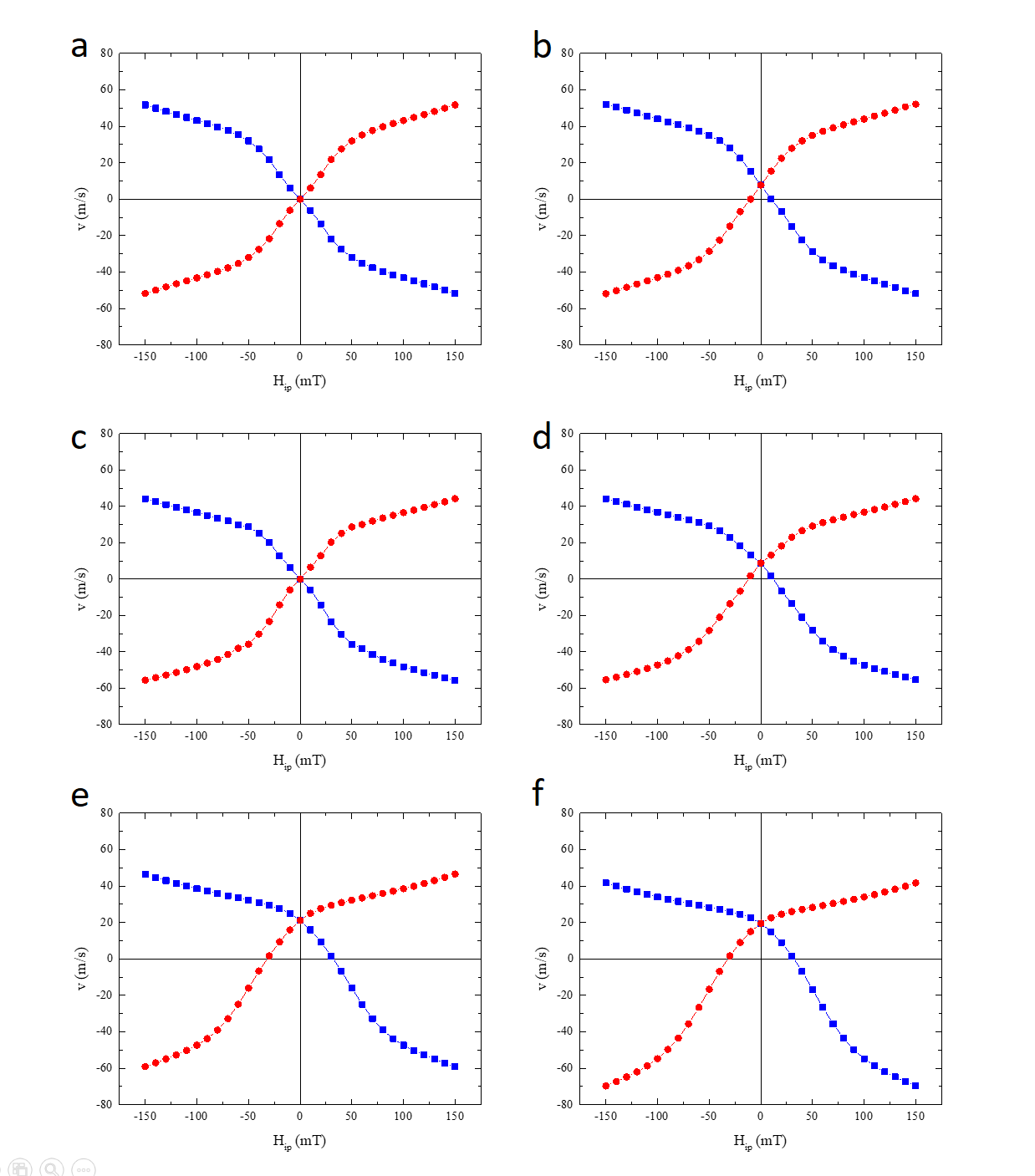


*Figure S10. Reversal of the asymmetry of the Current Induced DW motion (j=2×10^11^A/m^2^)* ***a****. H_DMI_ = 0, α_1_ = 0.2, α ^µ^_2_ = 0.* ***b****. H_DMI_= 10.4 mT, α_1_ = 0.2, α ^µ^_2_ = 0* ***c****. H_DMI_ = 0, α_1_ = 0.2, α ^µ^_2_ = 0.05.* ***d****. H_DMI_ = 10.4 mT, α_1_ = 0.2, α ^µ^_2_ = 0.05,* ***e****. H_DMI_ = 30 mT, α_1_ = 0.2, α ^µ^_2_ = 0.05,* ***f****. H_DMI_ = 30 mT, α_1_ = 0.2, α ^µ^_2_ = 0.1*

*
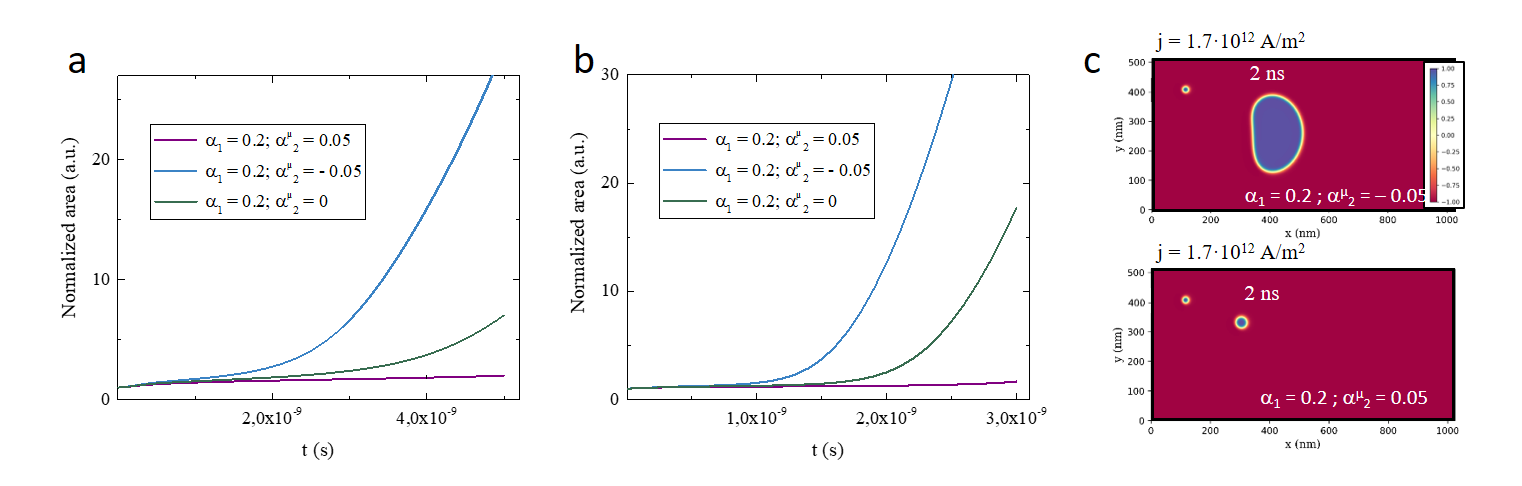
*

*Figure S11. Effect of the chiral damping on the Skyrmion dynamic stability.* ***a****. Evolution of the area of a dipolar stabilized skyrmion (seen in figure 5 of the main text) as a function of time for different chiral damping scenarios at 1.0×10^12^ A/m^2^ .* ***b****. Area of a smaller, skyrmion stabilized by DMI (seen in figure 5 of the main text) as a function of time at 1.7×10^12^ A/m^2^.* ***c****. Micromagnetic simulation of the deformation of the small topological skyrmion for different chiral damping scenarios.*

In order to evidence the effect of the chiral damping on the SK stability we stabilize skyrmions with an equilibrium diameter of 40 nm and we simulate the effect of the SOT as a function of the current density for different values of damping.

The first effect of chiral damping is to provoke a variation of the SK velocity. Second, the damping influences the distortion of the skyrmion’s circular shape. This is in agreement with the 2D velocity charts (Figure 5) calculated using the *q-φ* model.

The magnitude of the distortion is established by the competing effect of the asymmetric DW velocity and SK energy. If the SK is not sufficiently stable or the velocity asymmetry is too large, the SK size can diverge. In order to quantify this effect we use the total SK size as an indicator of the stability. In Figure S11 we plot the time evolution of SK size for a “large” dipolar stabilized SK as well as for a “small” SK stabilized by DMI. The majority of experimental studies realized at room temperature until now use the dipolar stabilized SKs. However, smaller skyrmions can be stabilized by using their topological protection. In our simulations, we were able to produce such “small” skyrmions, by slightly increasing the exchange coupling ($A={12\times10}^{-12} Jm^{-1}$). We confirm that in this case too, the chiral damping has a strong influence on the stability (Figure S11).

**References**

1. Thiaville, A., Garcia, J. M. & Miltat, J. Domain wall dynamics in nanowires. **245**, 1061–1063 (2002).

2. Slonczewski, J. C. DYNAMICS OF MAGNETIC DOMAIN WALLS. **5**, 170 (1972).

3. Schryer, N. L. & Walker, L. R. The motion of 180 ° domain walls in uniform dc magnetic fields. **45**, 5406 (1974).

4. Thiaville, A., Rohart, S., Jué, É., Cros, V. & Fert, A. Dynamics of Dzyaloshinskii domain walls in ultrathin magnetic films. *Europhys. Lett.* **100**, 57002 (2012).

5. Akosa, C. A., Takeuchi, A., Yuan, Z. & Tatara, G. Theory of chiral effects in magnetic textures with spin-orbit coupling. *Phys. Rev. B* **98**, 184424 (2018).

6. Jué, E. *et al.* Chiral damping of magnetic domain walls. *Nat. Mater.* **15**, 272–277 (2016).

7. Boulle, O., et al. "Current induced domain wall dynamics in the presence of spin orbit torques." *Journal of Applied Physics* **115**.17, 17D502 (2014).

8. Kim, J. Von. Role of nonlinear anisotropic damping in the magnetization dynamics of topological solitons. *Phys. Rev. B* **92**, 014418 (2015).

9. Abo, G. S., et al. "Definition of magnetic exchange length." *IEEE Transactions on Magnetics* **49**.8 4937-4939 (2013)

10. Vansteenkiste et al. “The design and verification of MuMax3”, AIP Adv. 4, 107133 (2014)

11. Mulkers et al., “Effects of spatially engineered Dzyaloshinskii-Moriya interaction in ferromagnetic films”, Phys. Rev. B 95, 144401 (2017)

12. Felix Büttner. et al." Field-free deterministic ultrafast creation of magnetic skyrmions by spin–orbit torques" *Nat. Nano.* **12**, 1040 (2017)
